# What Is Teamwork? A Mixed Methods Study on the Perception of Teamwork in a Specialized Neonatal Resuscitation Team

**DOI:** 10.3389/fped.2022.845671

**Published:** 2022-04-14

**Authors:** Krystyna Ediger, Marghalara Rashid, Brenda Hiu Yan Law

**Affiliations:** Department of Pediatrics, University of Alberta, Edmonton, AB, Canada

**Keywords:** infant, newborn, neonatal resuscitation, teamwork, communication barriers

## Abstract

**Introduction:**

Neonatal resuscitation is a team-based activity involving many decisions and tasks. Non-technical factors, such as teamwork, are increasingly recognized as impacting how well-neonatal resuscitation is performed, and therefore influencing infant outcomes. Prior studies on teamwork in neonatal resuscitation have focused on quantification of teamwork behaviors, or the effects of team training. This study aimed to explore healthcare providers' own perception of teamwork in this specialized environment to identify perceived barriers and facilitators to effective team functioning.

**Methods:**

This single-center exploratory sequential mixed methods study used two phases. First, semi-structured interviews were conducted, and thematic analysis used to identify themes. Subsequently, interview data informed the development of quantitative surveys to explore selected themes in the wider team.

**Results:**

From ten semi-structured interviews, seven themes were identified including: (1) Team Composition, (2) Effective Communication, (3) Team leadership, (4) Hierarchy, (5) Team Training, (6) Debriefing, and (7) Physical Environment. Perceived teamwork facilitators include role assignment, familiarity, team composition, talking out loud to maintain shared mental models, leadership, and team training. Perceived barriers included time pressures, *ad hoc* team, ineffective leadership, and space limitations. Selected themes (Communication, Speaking up, Hierarchy, and Leadership) were further explored via electronic surveys distributed via email to all members of the resuscitation team. There were 105 responses; a response rate of ~53%. All respondents agreed or strongly agreed that speaking up is important; however, not all felt comfortable doing so. Neonatal fellows (14%) and nurses (12%) were most likely to report not feeling comfortable speaking up. All respondents agreed that team communication is important to an effective resuscitation. Most respondents (91.5%) agreed that a hierarchy exists within the team; 54.3% believed that hierarchy positively contributes to effective resuscitation. All respondents agreed or strongly agreed that having a clearly defined leader is important in delivery room resuscitations. Ineffective leadership was the most cited reason for poor team communication.

**Conclusions:**

In this mixed methods study of perceptions of teamwork within a specialized, multi-disciplinary neonatal resuscitation team, communication, hierarchy, and leadership were positively perceived and facilitates teamwork. However, even in this environment, some nurses and trainees expressed discomfort with speaking up.

## Introduction

A team can be defined as a group of people with complementary and overlapping skills, working together to achieve common goals (1). Teamwork is one of the most effective work forms, providing better results than individual efforts (1). In healthcare, effective team functioning has been shown to decrease medical errors, decrease delays and maximize patient safety (2). Working effectively within a team also benefits healthcare workers, creating a shared purpose that fosters social connectedness and a sense of belonging, leading to increased job satisfaction and reduced burnout (3).

The neonatal intensive care unit (NICU) exemplifies a complex, high acuity healthcare environment dependent on teamwork and inter-professional collaboration. Delivering high-quality care to medically complex infants requires a cohesive team that collaborates and communicates well (4). Neonatal resuscitation is a key task in NICU care; in fact, ~10% of infants will require some help at birth, with 1% needing more extensive resuscitation such as endotracheal intubation and chest compressions (5). The risk of needing resuscitation at birth increases with risk factors such as prematurity and congenital anomalies; however, neonatal resuscitations are often unanticipated. As many tasks need to be coordinated during neonatal resuscitation, effective teamwork is essential. International guidelines such as the Neonatal Resuscitation Program (NRP) provide a standardized, evidence-based approach to resuscitation of newborn infants (6). Recently, NRP has increasingly emphasized the importance of non-technical factors, such as teamwork, recognizing its impact on neonatal resuscitation effectiveness; neonatal outcomes after resuscitation have been shown to benefit from collaboration and teamwork (4, 7).

There are different approaches to teams for neonatal resuscitation, depending on contextual factors such as location, availability of personnel, and anticipated acuity (8). While healthcare professionals (HCPs) are often highly trained in neonatal resuscitation, there can still be challenges in optimizing the function of neonatal resuscitation teams as a cohesive whole. Resuscitations can be stressful and unanticipated, with little time for preparation prior. Team members may not be consistent and may work together infrequently. Team size may vary depending on resuscitation complexity. New members, including trainees (e.g., resident doctors, nursing students, new hires) need to be integrated into this team frequently.

The importance of inter-professional teamwork in health care is well-documented in the health sciences literature. In 2000, the Institute of Medicine's seminal report *To Err Is Human: Building a Safer Health System* detailed the high rate of preventable medical errors, many of which are the result of dysfunctional or non-existent teamwork. Subsequently, through an examination of quality care and patient safety, it was shown that teamwork is essential to the provision of healthcare (9). Since, teamwork among HCPs has been found to improve patient outcomes in several settings. In the emergency room, team communication and collaboration are crucial for improvements to patient flow processes (10) and teamwork is seen a positive and effective construct in areas including resuscitation, patient outcomes and staff satisfaction (11). Emergency room nurses identify team communication, collaboration and leadership as critical elements of team effectiveness (11). Studies in Intensive Care Units (ICU) have reported reduced morbidity and mortality rates (12), lower incidence of medical errors and decreased delays in treatment (2) when health care teams function effectively.

The unique challenges neonatal resuscitation poses to effective teamwork have mostly been studied using quantitative methods. Thomas et al. quantified teamwork behaviors during neonatal resuscitations to identify relationships between observed teamwork behaviors and errors (13). Other studies on teamwork during neonatal resuscitation used observational metrics to quantify teamwork (14, 15). Finally, studies have been undertaken to assess the ability of team-training to improve quantitative teamwork measures (16).

To our knowledge, there are limited mixed methods studies examining team member perceptions of their own teamwork at a high-risk perinatal center. Prior studies have focused on NICU nurses (17), or teamwork in the NICU in general (18, 19). Other qualitative studies addressing resuscitation teamwork in the immediate post-natal period have been in low- or medium-income countries targeting basic neonatal resuscitation (20–22). Understanding healthcare providers' own perceptions of facilitators and barriers to successful teamwork could help direct education and quality improvement efforts to optimize team functioning. Exploring these perceptions using a mixed methods approach allows for rich and nuanced data, coupled with the ability to explore attitudes of the larger team in greater breadth and depth (23). Therefore, using an exploratory sequential mixed methods design, incorporating interviews and electronic surveys, we aim to explore how members of a specialized neonatal resuscitation response team conceptualize teamwork and team identity, as well as identify barriers and mediators to effective teamwork in this highly stressful, specialized, and unpredictable environment.

## Methods

### Study Design and Setting

This study used an exploratory sequential mixed methods (24, 25) approach with two phases. The initial phase consisted of descriptive qualitative methods to identify key themes, followed by a second phase that used quantitative surveys to further explore selected themes in a wider participant group. Data was obtained from the members of the multidisciplinary neonatal Resuscitation-Stabilization-Triage (RST) team at a regional perinatal referral center (Royal Alexandra Hospital) with a level 3 NICU, located in Alberta, Canada (8). The study was approved by the Health Research Ethics Board (Pro00088361).

### Participants and Sampling

The RST team consists of neonatologists, neonatal fellows (resident physicians training to be neonatologists), neonatal nurse practitioners (NNPs), advance practice transport nurses (TNs), registered nurses (RNs), and registered respiratory therapists (RTs), providing expert resuscitation to infants in distress after birth. Team composition varied depending on the complexity of the resuscitation and ranged from nurse-led, basic resuscitation team (TN+RN), to a large team composed of all disciplines. Team members receive neonatal resuscitation training via the Canadian NRP framework every 2 years and attend mandatory RST team update sessions annually. While associated with a pediatric training program, pediatric residents rotate for a total of only 8 weeks in this unit and were not considered core members of the RST team; thus, they were excluded from participation.

All RST team members were invited to participate through email and posters on the unit. For Phase 1, members of the RST team were recruited using purposeful sampling, with considerations for participants' discipline, experience, and role within the RST Team. After an initial email invitation was sent, interested participants were prioritized to ensure that all disciplines were represented in the first five interviews. Subsequently, upon examining our data and the participant's self-reported years of experience, a second email was sent, and we then targeted interested potential participants to include a learner (neonatal fellow), more junior team members (newer transport nurse, newer respiratory therapist), as well as a senior neonatologist. This ensured that both experienced and less experienced team members were included. Written informed consent was obtained from all interview participants. For Phase 2, all RST team members were invited to complete an electronic survey via email and study posters posted in the NICU. Study emails were sent by the RST team coordinator, who had no supervisory role and was not involved in the study. To minimize possible coercion, neither the study posters nor the invitation emails included the name of one researcher (BL), who was felt to have a potential supervisory role for some participants. Two reminder emails for survey completion were sent at 1 and 2 months after initial email. Consent was implied for submission of anonymized survey.

### Data Collection

Individual interviews were completed between July and September 2020. Interviews lasted approximately 60 min. Interviews were based on a semi-structured interview guide developed by the research team with expertise in qualitative research (MR), neonatal resuscitation (KE, BL), and human factors (BL) (Appendix A). This interview guide was intended to be as open-ended as possible without reference to existing teamwork models or known barriers and facilitators. Interviewers (KE, MR) were free to adjust their interview based on participant responses. Neither interviewer (KE, MR) had a supervisory role with the participants and one (MR) did not know the participants prior to the study. One researcher (BL) is an attending neonatologist with a possible supervisory role with some of the participants; thus she did not participate in the interviews. Given social distancing guidelines during the time of the COVID-19 pandemic, interviews were conducted via ZOOM videoconferencing (Zoom Video Communications Inc., San Jose, California) with field notes taken by the interviewers (KE, MR). Interviews were audio-recorded then transcribed verbatim using a combination of automatic electronic transcription (rev.com, San Francisco, California) with subsequent manual checking (KE). The research team (KE, MR, BL) met regularly during this phase to review data after individual data coding. Interviews continued until data saturation was reached, specifically, when no new themes were identified and with repeated recapitulation of themes previously seen.

Data from interviews informed the development of survey questions. Quantitative survey responses were ranked on a 5-point Likert scale. In addition, the survey allowed free text responses for each thematic category. This survey was sent via secure email link to all members of the RST team (*n* = ~200 potential participants). Online survey responses were anonymously collected on the University of Alberta's secure REDCap (Research Electronic Data Capture) database. Survey responses were collected over a 3-month period from February to May 2021.

### Data Analysis

Data analysis for Phase 1 occurred simultaneously with data collection. Analysis included reading the transcripts of all interviews and highlighting significant statements about the phenomena being studied. Thematic analysis was conducted to analyze the qualitative data. Application of a descriptive coding scheme assisted in identifying common themes in the experiences of various members of the resuscitation team. Each researcher first familiarized themselves with the transcripts through repeated readings, then individually coded the transcripts either with software (NVivo 12, QSR International Ltd. Burlington, USA) or paper coding. Researchers met several times during data collection to group codes into potential themes and subthemes. Differences in coding were debated to arrive at a consensus. The team then finalized the themes and subthemes as described in the results. To maintain trustworthiness, we used following strategies proposed by Lincoln and Guba (7): (1) credibility, (2) transferability, and (3) dependability and confirmability. To establish credibility, our team incorporated peer debriefing through regular research meetings to review our findings and reach consensus about decisions regarding recruitment, data collection and data analysis. Transferability refers to how relevant a study is to other settings and people. Study transferability is demonstrated by providing a “thick” (comprehensive) description of the study's methods, sample, and findings. Dependability and confirmability entailed keeping an audit trail of all decisions made during our study. Throughout the study, each team member reflected on their own judgments, beliefs, and feelings to minimize the effects of our biases, motives, and interests during the conduct of the study. In addition, having this form of reflexivity from the beginning to the end of the study played a vital role in increasing the overall quality of the study outcomes.

Quantitative survey data were summarized with descriptive statistics, using mean (Standard deviation, SD) and median (Interquartile range, IQR) for continuous parametric and non-parametric data, respectively. Data were compared using Student's *t*-test for parametric and Mann-Whitney *U* test for non-parametric comparisons of continuous variables, and Fisher exact for categorical variables. *P*-values were 2-sided and *p* < 0.05 was considered statistically significant. Statistical analyses were performed with SPSS Statistics for Macintosh, Version 27.0 (Armonk, NY: IBM Corp). Qualitative survey data (i.e., free text responses) were compared against themes identified in Phase 1 and incorporated where appropriate.

## Results

### Themes

**Figure 1:**
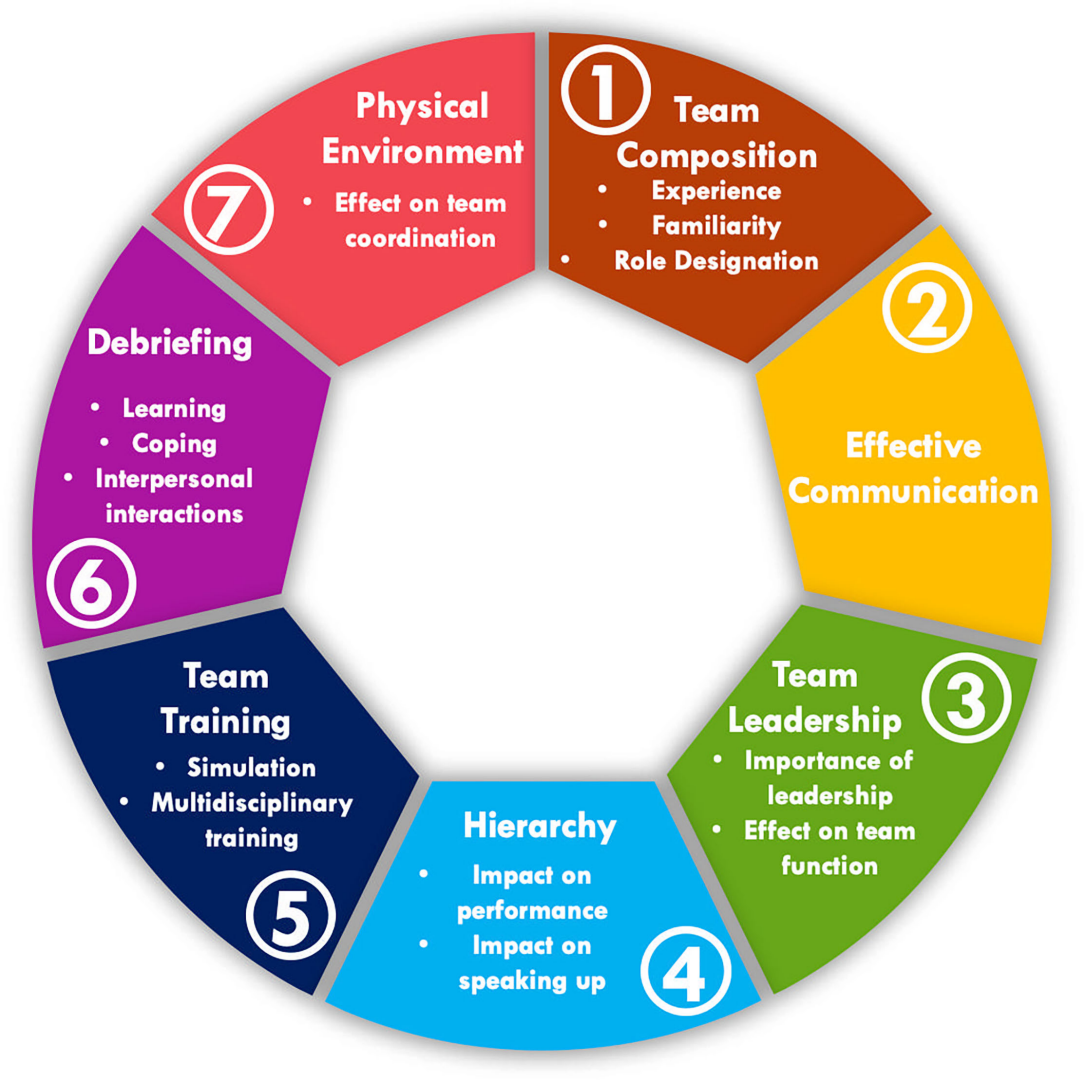
Themes and subthemes identified.

Ten participants were interviewed, representing all disciplines. Participants had a range of clinical experience (median 9.5 years, IQR 4.5–23) (Table 1). Seven themes were identified including: (1) Team composition, (2) Effective communication, (3) Team leadership, (4) Hierarchy, (5) Team training, (6) Debriefing, and (7) Physical Environment (Figure 1).

**Table 1:** Participant demographics.

Phase 1: semi-structured interviews	Discipline *n* (years of experience)	Neonatologist Neonatal nurse practitioner Neonatal fellow Transport nurse Nurse Respiratory therapist	2 (7 and 38 years) 2 (18 and 37 years) 1 (5 years) 2 (5 and 5 years) 1 (12 years) 2 (2 and 23 years)
	Gender (%)	Male (%) Female (%)	30% 70%
	Years of Neonatal Resuscitation Experience		Median 9.5 IQR 4.5–23
Phase 2: surveys	Discipline	Neonatologist Neonatal nurse practitioner Neonatal fellow Transport nurse Nurse Respiratory therapist	8 (87.6%) 21 (20.0%) 7 (6.7%) 11 (10.5%) 33 (31.4%) 25 (23.8%)
	Gender	Male (%) Female (%)	11.4% 88.6%
	Years of Neonatal Resuscitation Experience		Median 12 IQR 6–18
	Years of experience with RST Team		Median 8.5 IQR 3–11

#### Theme 1: Team Composition

Participants noted the importance of team composition on the effectiveness of teamwork, particularly highlighting the value of experience, familiarity, and role delegation. Explicit role assignment in the context of a multi-disciplinary team with overlapping technical skills was seen as particularly important:

“*I think about multidisciplinary and I think every individual has an important role to play and they should be separate. Especially with a limited number of people at a resus, it has to be clearly defined who's going to do what so that there's less overlap.” (Interview #5, Respiratory Therapist)*

However, there can be fluid transitions between formal and informal role assignment, based on each team member's experience and team members' familiarity with one another:

“*So as the team becomes more experienced and as a team, you recognize all the roles that need to be done. If it's not done, you just pitch in. So even though the roles are assigned at the beginning, you know, other people can do other things…like [people] are more aware of what may need to be done.” (Interview #6, Nurse Practitioner)*

Team composition and role delegation was insufficient for effective teamwork; some participants reflected on the importance of planning and working together to develop a shared mental model.

“*A shared mental model to me means that not just that we're all trying to get the same thing done, but we all understand one another's role in getting it done. So it isn't just we're all going to resuscitate the baby, it's knowing who's going to do what and making sure that we're adapted to that situation because otherwise we're just a group of people doing separate tasks, we're not a team. So to me, it's about a common understanding, common communication, common training, so that we understand one another's roles.” (Interview #10, Neonatologist)*

Finally, time pressures and the nature of an *ad hoc* team makes teamwork challenging, and increases the importance of clear communication to ensure appropriate role delegation and workload distribution:

“*I think it's challenging because you're always working with different people… So as much as you work with those people all the time, it's still different individuals in that group from day to day and people have their own styles, I guess, or methods. So I think just finding what works within that particular group of people for each baby makes it interesting always.” (Interview #4, Nurse)*

“*It's an initial plan of going through the roles, even making a plan to know if the resuscitation goes this way, what to do, what our plan is. If this resuscitation goes another way, what our plan is. Kind of just an idea of different scenarios that could happen and kind of going through as a team so we're all on the same page, essentially.” (Interview # 7, Neonatal Fellow)*

#### Theme 2: Effective Communication

Participants identified effective and efficient communication to be important to both technical performance and teamwork. Communication is seen as a key aspect of on-the-job training for junior members of the team, as well as a vital patient safety tool:

“*So I think one of the best markers of a good resuscitation team is communication. Because then even if there is a lack of knowledge or experience in some members at least then they're being kept up to date and then they're learning about what's going on and they learn from that experience too. And then the patient's safety is maintained because no one's really missing a huge thing that's going on because there's always people kind of mentioning once a while what's happening.” (Interview #1, Neonatologist)*

Many participants highlighted the importance of team members “talking out loud.” Team members who verbalize clearly about what they are doing and what actions are occurring, assist the team in maintaining situation awareness during the resuscitation:

“*If I'm doing MR.SOPA (mnemonic for troubleshooting steps for mask ventilation), if I say those words out loud and the leader was standing behind me they know exactly what I'm doing and so they don't have to ask, you don't have to go down that path twice.” (Interview #5, Respiratory Therapist)*

Participants noted that when the team leader verbalizes the direction of the resuscitation and ongoing plans, this helps the team understand the shared mental model:

“*Communication comes in… making sure everyone is aware of the situation between what happens with the newborn at the time and what needs to be done and make sure people know kind of what they need to know so they can make decisions.” (Interview #7)*

However, verbalizations can be disruptive:

“*Of course, a tricky balance. Honestly, you don't want too many people talking and too much noise, but I think it's a fine balance.” (Interview #1, Neonatologist)*

#### Theme 3: Team Leadership

According to our participants, teamwork in neonatal resuscitation is strongly influenced by team leadership. The key roles played by the team leader include maintaining situational awareness, establishing clear communication, and providing guidance:

“*I think essentially you're just guiding what the resuscitation, how the resuscitation is going to go. You're telling people what needs to be done. You're telling people you know, what needs to happen for the best outcome for this infant. And you're kind of guiding all members of the team to help resuscitate that baby.” (Interview #1, Neonatologist)*

“*What I feel makes them good is… they have that calm and good demeanor. Even the stressful situations, they keep their calm like, “Okay. Well, let's do this. Let's do this. Progress to that.” And like I said, they kind of talk out their resuscitation steps. So they verbalize what they're doing so that everyone kind of knows how the resuscitation is progressing... And that kind of also gives us some idea like, “Okay, so what's the next step? Is there something we should be doing also if they verbalize it and then they miss a step?” Then we can like, “Okay, did you want to try this before we move on?” So it's that communication. It's that calmness, that knowledge, honestly.” (Interview #7, Neonatal Fellow)*

In a more general sense, the team leader might positively or negatively affect team functioning via the “tone” or “vibe” they set for the team:

“*I think it ultimately comes down to the leader. I think the other members of the team can contribute to what the general vibe is in the room or how, or things are happening as a group. But I think the leader definitely can play a big part in setting the tone for that. Even if other people are feeling stressed or pressure or, you know, frustration, I think that can contribute in the dynamic, but less so than the leader perhaps.” (Interview #4, Nurse)*

Some participants expressed their opinion that different situations call for different leadership styles, and the ability of a leader to adapt is important:

“*There's different leadership styles and different circumstances call for different leadership styles, right. You know, sometimes when you are in a resuscitation, you might have to be that more directive type leader. It's not as collaborative as if you were sitting down in a room where, you know, like let's solicit everyone's feedback. At the same token when things aren't going well, and you can't really put your finger on why isn't this going well, what am I missing soliciting that collaborative feedback is important. So I think it just depends on that situation and being able to be there is the word for you more of a “situational leader.”” (Interview #2, Nurse Practitioner)*

#### Theme 4: Hierarchy

Hierarchy refers to perceived or actual power differences between team members based on professional designation, amount of experience and/or other factors. In a multi-disciplinary team structure with different roles and experience, an inherent and natural hierarchy is acknowledged and accepted:

“*I think there has to be a hierarchy, right? Because that's just kind of how it all works. Like I'm not the one making the decision of this is what we're doing for this patient, this is the things that need doing, this is why I'm deciding what needs to (be) done. We all have different backgrounds of knowledge and different training and experience and different responsibility. Like it's ultimately not up to me what we decided to do and it's up to me to help do it and to maybe provide my input or opinion, but that's not my responsibility. Like I will advocate for the patient or my thoughts on what is maybe happening. But also I will be receptive to, “this is why I'm doing this” or “just do this please”. So I think there is a hierarchy because that responsibility is different for each role.” (Interview #4, Nurse)*

The impact of hierarchy within the team on performance and on speaking up was highlighted throughout our interviews. Many participants felt that the presence of a hierarchy within the team was necessary for effective team functioning:

“*I mean obviously there's a hierarchy of experience. Some people are much more experienced than others. There is a hierarchy in terms of you do have to have leadership in the team. Somebody has to bring people together, so hierarchies are important in a team, but to me everybody brings value. And I still come back to the fact that the team trains its leader, so a leader is a servant of the team, not just a leader. It's like a politician they're a servant of the people, they're not just- they're not just a leader.” (Interview #10, Neonatologist)*

An inherent hierarchy and deference to disciplines that usually fill the leadership role (NNPs, physicians) was not necessarily seen as an impediment to open communication, specifically the ability for team members to “speak up” when they have questions, suggestions, or to point out errors:

“*There is in some senses a hierarchy because again, there are skills and things that I can do that not everyone can do. Like if we need like running a code, I think for the most part, we defer to the housestaff (NNPs and fellows). So whether it's an NP or a fellow to lead the code or the staff, if they're there. And I think that's just naturally how, how it happens. From my experience … I've never felt as though I am superior to the people who are not leading the code as far as that goes. So it was like, so as far as like hierarchy, if you want to say like the leader is like on top. Sure. But like at the same time, like I would be sunk without my team and I'm very aware of that. And so like, I don't ever look at it as like I'm in charge and you guys need to listen to me kind of deal. And like, it feels more like, again, like a team dynamic as far as I go.” (Interview #8, Respiratory Therapist)*

“*Hierarchy is necessary to have someone making the decisions. The leader, however should listen to all team members. Hierarchy as relates to the Neo (neonatologist) or Fellows or NPs in charge can be negative if they are not decisive, are not current with latest information or will not listen to other team members concern.” (Survey #37, Nurse Practitioner)*

“*I value the experience and knowledge of my team members. Often if I feel there is something I would want to speak on up, someone who is more experienced has already done so. With more experience, I think I'd become more comfortable in speaking up.” (Survey #24, Nurse)*

#### Theme 5: Team Training

Team training, specifically simulation and multi-disciplinary practice, was identified as an important means of team preparation for both routine and complex resuscitations:

“*I think the big thing would be able to prepare routinely. Sometimes you can't prepare when it's something that's urgent but getting more into the routine of talking through a situation or even before a complex delivery doing like a simulation pre delivery. I think simulation is a big thing that I would think is important to maintain skillset. Not even necessarily before a complicated delivery, just on a routine basis.” (Interview #1, Neonatologist)*

Simulation training was identified by multiple participants as a safe, low risk strategy to build team familiarity, identify strengths and weaknesses of each individual team members, and develop shared mental models:

“*I think it's helpful to have that kind of simulated environment where you can a get a sense of like how to actually do things without having that worry or that pressure like to necessarily have to get everything perfect first go round. And then also it helps you get an idea of like, okay, what are the strengths of the people that I'm going there with these people I've never actually worked with before? Like what are you able to do? How, how comfortable are you with compressions? How comfortable are you with intubation in a crash situation? How comfortable are you with the algorithm and getting a sense of like, where are the little hiccups that we could like anticipate the problems may be later down the road. I've always found simulation really, really helpful.” (Interview #8, Respiratory Therapist)*

#### Theme 6: Debriefing

Participants discussed how debriefing of resuscitation events assists with learning, coping, and interpersonal interactions, both after critical and more routine events. Debriefing is seen as highly important, but often occurring in more informal settings.

“*I think that there can be informal and formal debriefings. So often walking on the way back from whatever people have talked. When there hasn't been able to be a formal sit down, people have talked outside of work, you know, connected. But I think it should be how...if we press the code button, everybody has to come no matter what they're doing. And I think that a debriefing, it could be more of a “this has to happen” kind of thing. Cause misinformation can happen.” (Interview #9, Transport Nurse)*

“*My thought on debriefing is that we tend to use it more when it feels like maybe things have gone poorly or we've gotten a poor outcome, not necessarily the process has gone poorly, but the outcome has been poor. That's what kind of like debriefing always seems to have like these negative associations with it, but I think maybe there are scenarios in which debriefing after like a good outcome or a good process would also still be helpful and has some utility, I guess. And then it's easier for us to identify like what it is that we are doing well.” (Interview #3, Transport Nurse)*

#### Theme 7: Physical Environment

Participants noted the effects physical environment may have on team coordination. Limitations in the physical space can negatively affect teamwork, and limited separation between the resuscitation team and “outsiders” (i.e., family members, other medical professionals) can be perceived as “distracting” the team:

“*I think that the most challenging resus (resuscitation) I've ever done was one that was done in the OR. And the reason it was done in there is they weren't really anticipating that it was going to be like a, a not great delivery. It was one that just the RT and the RN had gone to. And then we got called like stat to come quickly to the bedside and they didn't really have the ability to bring him out. And so, like the challenge with that is that you have a very limited space and also a very present audience in terms of like the mom, the obstetrician, the anesthetist, like, and at one point the anesthetist was like over my shoulder, like his head is like right here. And again, like that can be really challenging cause there are a lot more distractions I feel as far as the environment goes.” (Interview #8, Respiratory Therapist)*

### Quantitative Survey Results

From the seven themes identified, four themes/subthemes were selected by the research team for further exploration. These focused on interpersonal interactions and included Speaking Up, Team Communication, Hierarchy, and Leadership. Themes were specifically selected to explore in a survey format as team members may have varying opinions depending on roles (Team Leadership, Communication), or may be perceived as more controversial (Speaking Up, Hierarchy). We specifically aimed to (1) determine prevalence of opinions amongst the entire RST team, (2) compare perceptions between team members in usual leadership positions (prescribers including NNPs and physicians), and team members in other roles (non-prescribers including TNs, RNs, and RTs), and (3) provide an opportunity for the RST team members not interviewed to offer their opinion on these subjects. For questions with categorical responses (e.g., most important role for a team leader), potential selections were derived from initial interview data with the ability to offer other answers if desired. After the themes were selected, the survey was developed by one researcher (KE), reviewed and edited by the other researchers (BL, MR), then pilot tested with three RST team members (neonatologist, respiratory therapist, nurse) prior to distribution.

There were 105 completed survey responses over a 3-month period, representing a response rate of ~53% (*n* = ~200 eligible to participate). Most (88.6%) identified as female. All disciplines were represented, including 33 RNs, 11 TNs, 25 RTs, 21 NNPs, 7 neonatal fellows, and 8 neonatologists. Prescribers (NNPs, neonatal fellows, and neonatologists), who are usually the team leaders in complex resuscitations, represented 34.3% of respondents. Most respondents were very experienced, with a median of 12 (IQR 6–18) years of neonatal resuscitation experience, and median of 8.5 (IQR 3–11) years as a part of the RST team (Table 1).

#### Speaking Up

All respondents agreed or strongly agreed with the statement “Speaking up is important.” However, not all felt comfortable doing so: more non-prescribers disagreed or strongly disagreed with the statement “I feel comfortable speaking up” than prescribers (16 vs. 5.5%, *p* = 0.211). Neonatal fellows (14%) and nurses (12%) had the highest proportion of responses not being comfortable; in contrast, no NNP or Neonatologist felt uncomfortable. When asked about their perceptions of other team members feeling comfortable speaking up, 16% of non-prescribers vs. 5.5% of prescribers disagreed or strongly disagreed with the statement “I think that other members of the resuscitation team feel comfortable speaking up during a resuscitation” (*p* = 0.211) (Table 2).

**Table 2:** Summary of survey results.

**Theme**	**Question**	**Responses**			
		**(% agree or strongly agree)**			
		**Overall**	**Prescribers^a^**	**Non-prescribers^b^**	**Comparison (*p*)**
Speaking up	I believe that speaking up during a resuscitation is important.	100	100	100	—
	I feel comfortable speaking up during a resuscitation.	80.9	91.7	73.9	0.259
	I think that other members of the resuscitation team feel comfortable speaking up during a resuscitation.	59.1	63.9	53.6	0.211
Team communication	I feel that team communication is important to an effective and efficient resuscitation.	100	100	100	—
	I feel that I communicate well in my role as a member of the RST team.	93.1.	86.1	95.7	0.119
	I feel that the RST team at the Royal Alexandra Hospital usually communicates well with each other during a resuscitation.	94.0	100	88.1	0.259
Hierarchy	I believe that a hierarchy exists within the RST team at the Royal Alexandra Hospital.	91.5	91	92	1.0
	I feel that having a hierarchy in the resuscitation team is important.	55.2	61.1	52.2	0.569
	I feel that a hierarchy in the resuscitation team contributes POSTIVELY to effective resuscitation.	54.3	55.6	54.4	0.250
	I feel that a hierarchy in the resuscitation team contributes NEGATIVELY to effective resuscitation.	26.4	19.4	31.9	0.669
Leadership	I feel that having a clearly defined leader is important in resuscitation events in the delivery room.	100	100	100	—
	We usually have a clearly defined leader during resuscitation events in the delivery room.	90.5	87.0	97.2	0.159

a *“Prescribers” include neonatologists, neonatal fellows, and nurse practitioners, who have prescribing privileges and are usually the team leaders of the resuscitation team during a resuscitation*.

b *“Non-prescribers” include nurses, advance practice transport nurses, and respiratory therapists*.

#### Team Communication

All respondents agreed or strongly agreed with the statement “I feel that team communication is important to an effective and efficient resuscitation.” Only one respondent (non-prescriber) felt that they did not communicate well in their role. Most respondents agreed or strongly agreed with the statement “I feel that RST team at the Royal Alexandra Hospital usually communicates well with each other during a resuscitation.” All disagree responses were from non-prescribers (6% of non-prescribers). When recalling an instance when communication did not go well, ineffective leadership was the most commonly selected reason (65%), followed by roles not defined (63%), and lack of time to prepare (52%), with other reasons including lack of experience of team members (47%), presence of other people outside of the resuscitation team (21%) (Table 2).

#### Hierarchy

Most respondents (91.5%) agreed or strongly agreed with the statement “I believe that a hierarchy exists within the RST team.” There was no difference in this perception between prescribers and non-prescribers (91 vs. 92%, *p* = 1.0) A majority (55.2%) agreed or strongly agreed with the statement “I feel that having a hierarchy in the resuscitation team is important,” with 54.3% agree or strongly agree that a hierarchy contributes positively to effective resuscitation (Table 2).

#### Team Leadership

When asked about team leadership, all respondents agreed or strongly agreed with the statement “I feel that having a clearly defined leader is important in resuscitation events in the delivery room.” Most (90.5%) respondents agreed or strongly agreed that “We usually have a clearly defined leader during resuscitation events in the delivery room.” When asked to select the top three qualities for a good team leader, 87% choose “Clear communicator,” 84% chose “Ability to remain calm,” 57% choose “Knowledgeable,” 29% chose “Experienced,” 18% choose “Approachable,” 13% chose “Well respected by team members,” “Organized” 8% and “Trustworthy” 4%. When asked about the most important function of a team leader, both prescribers (69%) and non-prescribers (67%) chose “Maintaining situational awareness” most frequently (*p* = 0.586), followed by “Decision making” (22 vs. 23%), and “Coordination” (6 vs. 10%) (Table 2).

## Discussion

To our knowledge, our study was one of the first to use mixed-methods to investigate the perception of teamwork within a specialized, high-risk neonatal resuscitation team from the viewpoint of the team members themselves. Our study recognized many factors that HCPs themselves identify as facilitators and barriers to effective teamwork in neonatal resuscitation. Perceived facilitators for teamwork include formal and flexible role assignment, team familiarity, multidisciplinary team composition, talking out loud to maintain shared mental models, effective leadership, and simulation team training. Perceived barriers including time pressures, *ad hoc* nature of the team, ineffective leadership, and physical space limitations. Some themes echo those identified in previous studies (17–19). Thomas et al. conducted focus groups and identified three main themes including providers factors, workplace factors, and group influences (19). More recently, Salih et al. conducted simulations for in-unit NICU emergencies (e.g., unintended extubation), then analyzed the post-simulation debriefings to identify perceived barriers and facilitators to effective teamwork (18). Some similar themes and subthemes were identified, such as speaking out loud, team composition and roles, leadership, synergy of the team. However, in contrast to these studies, our study focuses on a specialized high-risk delivery room resuscitation team, and augmented thematic analysis with a follow-up quantitative surveys targeting topics of interpersonal interactions (Speaking Up, Team Communication, Hierarchy, and Leadership). Overall, despite challenges identified, participants in our study had a positive outlook on personal interactions within this multi-disciplinary neonatal resuscitation team.

In our study, HCPs reported the importance of composition of the team, both in terms of professional designation as well as level of experience, as a key factor in effective team functioning. Existing literature on team dynamics highlights the importance of careful team formation and composition (26, 27). In teams with stable membership, effective communication, and coordination structures, familiarity with each other's knowledge and mutual trust can be developed over time (28). As is common in critical care medicine, the RST team at the Royal Alexandra Hospital is composed of numerous members of different specialties, who form *ad hoc* teams based on schedules, rotations, and clinical need. Due to varying team composition, significant barriers can be faced by such teams, as they often “lack opportunity to develop a team identity, shared mental models, and trust” (29). These challenges were identified in our study, with HCPs recognizing the importance of pre-briefing, role delegation and shared mental models to maximize effective team functioning. There was also a recognition that as team members worked together more often, a level of trust and familiarity was formed, improving teamwork. Thus, strategies to improve neonatal resuscitation teamwork should include both structural/process changes (e.g., mandated pre-delivery briefings, checklists, cognitive aids, “best practice” guidelines) and team building (e.g., multidisciplinary simulation training, education and quality improvement initiatives targeting interpersonal communication and positive work culture) working in tandem. Quality improvement initiatives using both process changes and education to facilitate team functioning have been demonstrated to improve short-term markers of resuscitation quality (30). Our study reinforces that even within a highly specialized team with a positive outlook on teamwork and communication, HCPs themselves identify potentially modifiable factors to further improve team function.

Our participants felt strongly that communication was important to effective and efficient neonatal resuscitation. This is in keeping with published literature that has found effective interpersonal communication to be fundamental to successful teamwork during neonatal resuscitation (27). Components of effective communication cited in the literature include information sharing, speaking up, and closing the loop (7, 31, 32). Our participants identified “Talking out loud” as a method of maintaining a shared mental model to facilitate teamwork. Strong team leadership was felt to be an important facilitator of good team communication, while lack of clear, effective leadership was seen to contribute to poor team communication. When asked to identify qualities of a strong leader, traits such as clear communicator, ability to remain calm, and knowledgeable were prioritized. Interestingly, traits such as experienced, approachable, well-respected by team members, and trustworthy were seen to be less imperative. This may speak to the experienced and specialized nature of the RST team itself, where the expectation is that all leaders are experienced and trustworthy, but where leadership and communication styles are more variable. While non-technical skills such as communication and team leadership has been increasingly emphasized in national neonatal resuscitation educational programs such as NRP (6), what constitutes “good team leadership” may not be universal and may be highly dependent on each unit's culture and team structure. These variations should be considered in the design of interventions to improve neonatal resuscitation teamwork in different cultural and resource settings.

Even though nurses often play a lead role within the team, both our interview and survey participants believe that a hierarchy exists within the resuscitation team. Interestingly, many identified this as a positive and necessary aspect of team function. However, while hierarchy was not stated explicitly as a barrier to teamwork, presence of a hierarchy, difference in experience, and leadership styles were noted to be potential barriers to speaking up. This finding might be explained by a perceived lack of psychological safety for some team members within the team's hierarchy. It has been shown that when teams operate in a manner that is democratic, supportive, and welcoming of questions and challenges, team members are likely to feel greater psychological safety and an increased level of comfort in speaking up and offering suggestions or challenges (33). In a multi-disciplinary team environment, speaking up can be particularly difficult for trainees (e.g., residents and fellows) as well as nurses, compared with attending physicians (34, 35), a finding reflected in our survey results. Psychological safety contributes to an environment where errors can be identified by any member of the team. However, in the time sensitive, high workload, and rapidly changing neonatal resuscitation context, there exists a balance between effective team leadership, efficient team functioning, and democratic team discussions. As voiced by one of our interview participants, “different circumstances call for different leadership styles”; adapting one's leadership style to the circumstances may be an important aspect of neonatal resuscitation leadership training.

Our study has several strengths. Our team operates at one of the busiest NICUs in Canada, serving more than 15,000 deliveries per year. Participants involved represented a wide variety of HCPs with varying levels of neonatal resuscitation experience, including trainees (neonatal fellows). Over half of eligible participants completed our survey, a strong response rate (36). Our study also has several limitations. This is a single center study, where participants are members of an experienced and specialized neonatal resuscitation team operating within a North American cultural context. Our center has unique professional roles that may not be present in other units (i.e., nurse practitioners, respiratory therapists, transport nurses) and operate in a high resource country with a public health system. Therefore, our results may not be generalizable to those working in smaller, less dedicated teams, or in settings with different resources, team structures, and hierarchies. Finally, we did not examine the relationship between HCPs perceived quality of teamwork, their perception of their resuscitation performance, and patient outcomes.

## Conclusion

In this mixed methods study of perceptions of teamwork within a specialized, multi-disciplinary neonatal resuscitation team, seven themes were identified, centered around interconnected issues of team structure, leadership and hierarchy, communication, and training. Perceived facilitators to teamwork include role assignment, familiarity, team composition, talking out loud, leadership, and team training. While communication, hierarchy, and leadership were positively perceived overall, some nurses and physician trainees expressed discomfort with speaking up. Other perceived barriers include time pressures, *ad hoc* nature of the team, ineffective leadership, and physical space limitations.

## Data Availability Statement

The original contributions presented in the study are included in the article/Supplementary Material, further inquiries can be directed to the corresponding authors.

## Ethics Statement

The studies involving human participants were reviewed and approved by University of Alberta Health Research Ethics Board (Pro00088361). Written informed consent was obtained from all interview participants. Consent was implied for submission of anonymized survey.

## Author Contributions

All authors were responsible for conception and design, collection and assembly of data, analysis and interpretation of the data, drafting of the article, critical revision of the article for important intellectual content, final approval of the article, and contributed to the article and approved the submitted version.

## Conflict of Interest

The authors declare that the research was conducted in the absence of any commercial or financial relationships that could be construed as a potential conflict of interest.

## Publisher's Note

All claims expressed in this article are solely those of the authors and do not necessarily represent those of their affiliated organizations, or those of the publisher, the editors and the reviewers. Any product that may be evaluated in this article, or claim that may be made by its manufacturer, is not guaranteed or endorsed by the publisher.
