# Supplementary material for: What Is Teamwork? A Mixed Methods Study on the Perception of Teamwork in a Specialized Neonatal Resuscitation Team
Source: Front Pediatr. 2022 Apr 14;10:845671. doi: 10.3389/fped.2022.845671 (PMC9046838; doi:10.3389/fped.2022.845671)
Supplement: Supplementary file 1 [file Table_1.DOCX]

**Supplementary Table 1:** Identified Themes and Supporting Quotations

| Themes | Quotations | Interview/Survey Response |
| --- | --- | --- |
| *Team Composition* | “...teamwork's essential. Mainly because of the number of things that need to be done at once, but also largely because the multiple perspectives and experience and specialty backgrounds really help to come together to address all the different aspects needed to care for that baby. So you'll have the lead person, which is usually a neonatal nurse practitioner or NICU fellow who will be typically in a high risk situation, managing the airway and kind of, or potentially leading the code itself or the resuscitation itself. So they have the overall medical background to be able to guide things. And then the skill set regarding airway and intravenous access in terms of umbilical lines. Then you have the nurses whose specialty is assessing clinically like air entry, things like that, putting monitors on, drawing up medications, getting medications ready if needed, and then doing many, many other things such as, you know, assisting with compressions, recording, talking to parents, doing lots of things like that.” | Interview 1 |
|  | “I think having the appropriate number of people is number one. I think having the, not only the appropriate number of people, but the people that are experienced and comfortable, and I know you always need to have new learners and you need to have those people there, but truly a good resuscitation or one that I can remember as being like a good resuscitation has experienced people that has that ability to anticipate what the next move is going to be, whether it's communicated or not.” | Interview 2 |
|  | “When we have like the appropriate number of people and roles have been designated and it's clear who's doing what it, it feels just really good after knowing that you've kind of dealt with a critical situation, but it's all good. I think generally speaking, it goes very smoothly.” | Interview 3 |
|  | “I think it's challenging because you're always working with different people, different practitioners from all the different disciplines. So you, as much as you work with those people all the time, it's still different individuals min that group from day to day and people have their own styles, I guess, or methods. So I think just finding what works within that particular group of people for each baby makes it interesting always. | Interview 4 |
|  | “I think about multidisciplinary, to start, and I think about every individual has an important role to play and they should be separate. Especially with a limited number of people at a resus, it has to be clearly defined who's going to do what so that there's less overlap. I think, the more efficient things are, probably the smoother things go. Even during setup I think it's important for everybody to say what they're checking or what they have checked because lots of times, especially if people arrive at staggered times, people will be checking stuff that's already been done when their time maybe could be used better priming lines or getting intubation supplies ready because somebody else may have already checked the t-piece and the FiO2 and the mask size and all of that. So I think it's a matter of everybody vocalizing what they're doing so it's not being duplicated or triplicated, particularly if it's an imminent delivery that we weren't expecting.” | Interview 5 |
|  | “So as the team becomes more experienced and as a team, you recognize all the roles that need to be done. If it's not done, you just pitch in. So even though the roles are assigned at the beginning, you know, other people can do other things like the nurses are more aware of what, what may need to be done.” | Interview 6 |
|  | “When everyone has their own roles during the resuscitation, it's good to know who has which skills and what your best role is and where you should be in the resuscitation.” | Interview 7 |
|  | “With teamwork, a lot of it I feel is important even before resuscitation. It's an initial plan of going through the roles, even making a plan to know if the resuscitation goes this way, what to do, what our plan is. If this resuscitation goes another way, what our plan is. Kind of just an idea of different scenarios that could happen and kind of going through as a team so we're all on the same page, essentially. And I think that's quite important. I've been in resuscitations where not everyone has kind of gotten the full plan that was made previously before they arrived. And then there's a little bit disconnect during resuscitation. So I think it's very important.” | Interview 7 |
|  | “Right. What comes into mind is, I mean to me the most important thing about a team is that we have a shared mental model, that we have a common understanding of what we're trying to do. So all the competencies that we teach, team competencies we teach in NRP and more come to mind when thinking about teamwork and neonatal resuscitation. A shared mental model to me means that not just that we're all trying to get the same thing done, but we all understand one another's role in getting it done. So it isn't just we're all going to resuscitate the baby, it's knowing who's going to do what and making sure that we're adapted to that situation because otherwise we're just a group of people doing separate tasks, we're not a team. So to me, it's about a common understanding, common communication, common training, so that we understand one another's roles.” | Interview 10 |
|  | “In my experience, the times when resuscitation felt uncomfortable for me were when someone new was learning to either "take the head" or learning to lead, in which communication was lacking between the learner and the teacher and the teacher, I feel, did not step in soon enough.” | Survey 9 |
| *Effective communication* | “So I think one of the best markers of a good resuscitation team is communication. Because then even if there is a lack of knowledge or experience in some members at least then they're being kept up to date and then they're learning about what's going on and they learn from that experience too. And then the patient's safety is maintained because no one's really missing a huge thing that's going on because there's always people kind of mentioning once a while what's happening. Of course, a tricky balance. Honestly, you don't want too many people talking and too much noise, but I think it's a fine balance”. | Interview 1 |
|  | “...also if the communication is there, people are saying, "let's plan for this", or 'can you get this ready just in case". So I think it's also a group dynamic to know why you might be trying to accomplish first.” | Interview 4 |
|  | “I think if there's clear communication between all the team members, right? If I'm doing MR.SOPA, if I say those words out loud and the leader was standing behind me they know exactly what I'm doing and so they don't have to ask, you don't have to go down that path twice.” | Interview 5 |
|  | “Communication part comes in, making sure everyone is aware of the situation between what happens with the newborn at the time and what needs to be done and make sure people know kind of what they need to know so they can make decisions.” | Interview 7 |
|  | “Communication is the first thing. Knowing your roles, if you have defined roles in good communication, it's often, it's a well oiled machine. So the communication would be like I said before, if you're noticing something that's not going well, if you're noticing something that can change and then communicating the effectiveness of the treatment, communicating whether you think you need more help or not. And then, so I think obviously communication is skills you can learn and perfect, but I think that often you can, anyone can do those skills if they need to, if you can communicate with them how to do it. But the, yeah, I think just anticipate anticipating each other's needs and communicating well would be teamwork to me.” | Interview 9 |
| *Team leadership* | “I think essentially you're just guiding what the resuscitation, how the resuscitation is going to go, right You're telling people what needs to be done. You're telling people you know, what needs to happen for the best outcome for this infant. And you're kind of guiding all members of the team to help resuscitate that baby. | Interview 1 |
|  | “But you know, there's different leadership styles and different circumstances call for different leadership styles, right. You know, sometimes when you are in a resuscitation, you might have to be that more directive type leader. It's not as collaborative as if you were sitting down in a room where, you know, like let's solicit everyone's feedback. At the same token when things aren't going well, and you can't really put your finger on why isn't this going well, what am I missing soliciting that collaborative feedback is important. So I think it just depends on that situation and being able to be there is the word for you more of a "situational leader." | Interview 2 |
|  | “I think it ultimately comes down to the leader. I think the other members of the team can contribute to what the general vibe is in the room or how, or things are happening as a group. But I think the leader definitely can play a big part in setting the tone for that. Even if other people are feeling stressed or pressure or, you know, frustration, I think that can contribute in the dynamic, but less so than the leader perhaps.” | Interview 4 |
|  | “I think... I love it when leaders talk through things because it helps me to understand what's happening and what's going through their heads, like, "Okay. We tried this and this isn't happening but it should," you know and ask questions of the group. "Am I missing anything? Is there anything you can think of why this didn't work," or whatever. | Interview 5 |
|  | “What I feel makes them good is that, like I said, they have that calm and good demeanor. Even the stressful situations, they keep their calm like, "Okay. Well, let's do this. Let's do this. Progress to that." And like I said, they kind of talk out their resuscitation steps. So they verbalize what they're doing so that everyone kind of knows how the resuscitation is progressing or at their pace. And that kind of also gives us some idea like, "Okay, so what's the next step? Is there something we should be doing also if they verbalize it and then they miss a step?" Then we can like, "Okay, did you want to try this before we move on?" So it's that communication. It's that calmness, that knowledge, honestly. Sometimes the experience, having people who've seen different types of resuscitations, different types of outcomes, to kind of lead you through a situation. Like I said, that communication is key so that, at least, to the rest of the team, knows what's going on and can kind of help go through the resuscitation with the team lead as well.” | Interview 7 |
|  | “I think there has to be a hierarchy, right? Because that's just kind of how it all works. Like I'm not the one making the decision of this is what were doing for this patient, this is the things that need doing, this is why I'm deciding what needs to done. We all have different backgrounds of knowledge and different training and experience and different responsibility. Like it's ultimately not up to me what we decided to do and it's up to me to help do it and to maybe provide my input or opinion, but that's not my responsibility. Like I will advocate for the patient or my thoughts on what is maybe happening. But also I will be receptive to, "this is why I'm doing this" or "just do this please". So I think there is a hierarchy because that responsibility is different for each role. And I think people perceive that differently also. And again, going back to your experience working with people, you might be more willing to take somebody's thoughts into consideration if you've worked with them a long time and trust their judgment and their experience, as opposed to somebody that you don't know what experience they have, or this is your first time doing this, is this your hundredth time doing this? So I think until you kind of have a better understanding of that, I mean, there's a hierarchy for a reason because everyone has their own responsibility in that situation, but you also are working as a unit. So I find for the most part, I think each person's input is valued and their opinion of what needs to happen next is valued or at least acknowledged.” | Interview 4 |
|  | “There is in some senses a hierarchy because again, there are skills and things that I can do that not everyone can do. Like if we need like running a code, I think for the most part, we defer to the housestaff. So whether it's an NP or a fellow to lead the code or the staff, if they're there. And I think that's just naturally how, how it happens. From my experience I've like, I guess from my perspective, I've never felt as though I am superior to the people who are not leading the code as far as that goes. So it was like, so as far as like hierarchy, if you want to say like the leader is like on top. Sure. But like at the same time, like I would be sunk without my team and I'm very aware of that. And so like, I don't ever look at it as like I'm in charge and you guys need to listen to me kind of deal. And like, it feels more like, again, like a team dynamic as far as, as far as I go.” | Interview 8 |
|  | “I mean obviously there's a hierarchy of experience. Some people are much more experienced than others. There is a hierarchy in terms of you do have to have leadership in the team. Somebody has to bring people together, so hierarchies are important in a team, but to me everybody brings value. And I still come back to the fact that the team trains its leader, so a leader is a servant of the team, not just a leader. It's like a politician they're a servant of the people, they're not just- they're not just a leader.” | Interview 10 |
|  | “I think it's important to have a hierarchy so that there is a clear leader, but I can see how this mindset may lead people to feel like they can't speak up or challenge the leader.” | Survey 5 |
|  | “It is important to speak up for the wellness of the babies if the resus lacks leadership or there is some part that makes me uncomfortable. It is also important to support and validate your team because of how stressful resus can be!” | Survey 8 |
|  | “A hierarchy can be positive or negative depending who is at the top of the hierarchy. In ineffective leader will often lead to problems/confusion in the resus. Sometimes this forces another person near the top of the hierarchy to intervene. Depending on their relationship, this can lead to tension between the team.” | Survey 14 |
|  | “I think it is important to put ego aside and advocate for the patient. It is very important to speak up.” | Survey 17 |
|  | “I value the experience and knowledge of my team members. Often if I feel there is something I would want to speak on up, someone who is more experienced has already done so. With more experience, I think I'd become more comfortable in speaking up.” | Survey 24 |
|  | “Hierarchy is necessary to have someone making the decisions. The leader, however, should listen to all team members. Hierarchy as relates to the Neo or Fellows or NPs in charge can be negative if they are not decisive, are not current with latest information or will not listen to other team members concern.” | Survey 37 |
|  | “I think hierarchy in terms of professional experience can be important, and issues arise when people resort to hierarchy based purely on professional designation. This is where I have run into issues in the past, of feeling uncomfortable to speak up when a fellow is in a learning position and I feel as though the neo should be providing them feedback and that is not being done. I do not want to overstep.” | Survey 42 |
|  | “I think "hierarchy" can be good and bad! In the situation with a strong leader, the hierarchy naturally makes them a leader and everything goes smoothly. In the opposite situation, it's disastrous! I believe leadership naturally falls on the undefined "hierarchy"... neo, to nnp/fellow, to TN, to most experienced RST.... making someone a leader, whether they are a "good" leader, or not!” | Survey 58 |
| *Team training (including simulation and multidisciplinary training)* | “I mean, I think the the big thing would be able to prepare routinely. Sometimes you can't prepare when it's something that's urgent, but getting more into the routine of talking through a situation or even before a complex delivery doing like a simulation pre delivery. I think simulation is a big thing that I would think is important to maintain skillset. Not even necessarily before a complicated delivery, just on a routine basis. But I'm biased because I'm trying to kind of be involved in that too, but I think that would facilitate more efficient resuscitation and learning because we only, we don't have, well, sometimes you have sick babies pretty often, but sometimes you have long stretches for weeks without a really sick baby. So how do you maintain the freshness in terms of your brain's ability to recall things in a reflexive manner?” | Interview 1 |
|  | “There's always a role for simulation. I think it...and, actually, to be very honest, people don't like it when you do it, but it's where they learn the most, is videotaped simulations and doing a debrief while we're doing it. People see things in themselves. But simulation without video is also very valuable for all things and I think it would be way to get maybe some of the less experienced people or even work with some of the newer RTs, put them at the "head of the bed" with an assistor and they have to sort of lead their way through it just to get more comfortable with all the MR.SOPA, the dry, stimulate, da, da, da, da, da.” | Interview 5 |
|  | “I think it's helpful to have that kind of simulated environment where you can a get a sense of like how to actually do things in the Alex without having that worry or that pressure like to necessarily have to get everything perfect first go round. And then also it helps you get an idea of like, okay, like what are, what are the strengths of the people that I'm going there with these people I've never actually worked with before? Like what are you able to do? How, how comfortable are you with compressions? How comfortable are you with intubation in a crash situation? How comfortable are you with the algorithm and getting a sense of like, where are the little hiccups that we could like anticipate the problems may be later down the road. I've always found simulation really, really helpful.” | Interview 8 |
|  | “I do think the more sims we can run as a multi- disciplinary team helps. I also think its helpful to address individuals about behaviors or specific incidents that were problematic. This shouldn't be done in a punitive manner, but I personally know that it helps to have feedback when you are learning, or when things don't go well. We need to stress that feedback isn't about criticism but about growth”. | Survey 30 |
|  | “As a new member of the RST team, my knowledge is directly linked to my experiences at work. Some things may take longer for people to have exposure to and it's important not to judge people based on their time in the role. I often feel judged for not knowing things, but it's not that I don't WANT to know them, I just haven't had the opportunity to learn them yet.” | Survey 43 |
| *Debriefing (the importance of debriefing of critical events to assist with learning, coping and interpersonal interactions)* | “Sometimes it's challenging because so many of the staff and nurses have to do so much after the resuscitation and so then you try to meet after, but sometimes there's no time, it's too busy and then the shift is over and everyone's gone. And I don't know how better to do that unless you take two or three minutes when there's a little bit of stability and quickly chat, but I mean, a quick debrief isn't ideal either. But I think that is improving compared to when I was in fellowship. Now when there' a code or a significant resuscitation happens, people almost automatically think about a debrief. And I think it'll become more and more commonplace that people will make time for it over time.” | Interview 1 |
|  | “I think debriefing becomes quite important afterwards that you can actually talk about the things that went on and why those types of behaviors may have come out of me or came out of them or, you know how we can move on from that. Get better at it, truly.” | Interview 2 |
|  | “My thought on debriefing is that we tend to use it more when it feels like maybe things have gone poorly or we've gotten a poor outcome, not necessarily the process has gone poorly, but the outcome has been poor. That's what kind of like debriefing always seems to have like these negative associations with it, but I think maybe there are scenarios in which debriefing after like a good outcome or a good process would also still be helpful and has some utility, I guess. And then it's easier for us to identify like what it is that we are doing well, and yeah.” | Interview 3 |
|  | “And you know, we're supposed to do a debrief afterwards, but it can be very difficult because if the baby survives, then you usually have a lot to do. So it's hard to break off and do a debrief when you're still really resuscitating the baby in a different space.” | Interview 6 |
|  | “Well, I think it's important, but like I say, I don't think it happens as much as it should because you know, shifts might change or something like that and you can't get these people together. And then the person doing the debriefing, it's not always helpful to have the team leader doing it. Cause perhaps the problems arise from the team leader and not, and not from other things. So, you know, in some respects it would be better to have a neutral person do the debriefing. And then I think people be more willing to actually come out with what they, what they felt.” | Interview 6 |
|  | “I think that there can be informal and formal debriefings. So often walking on the way back from whatever people have talked. When there hasn't been able to be a formal sit down, people have talked outside of work, you know, connected. But I think it should be how...if we press the code button, everybody has to come no matter what they're doing. And I think that a debriefing, it could be more of a "this has to happen" kind of thing. Cause misinformation can happen, which is also something that the CISM team often has to deal with. Is people around the unit are hearing misinformation about what happened in a situation and then they're feeling distressed on that and they weren't even involved in the situation. So yeah, I think the, the things that are done well in our, when we have had formal debriefings are, you know, not shaming anybody talking about how we could do or how we did things well, what could improve next time and, you know recognizing and appreciating everybody on the team for what they did do, because I think sometimes people think they didn't do a good job or feel under appreciated.” | Interview 9 |
|  | “I think that debriefing is important. I think that briefing is important, it's really good especially when you have new people for them to know what to expect. So to tell them, "This is how it's likely to go." So you're briefing them. Sometimes you go through rare procedure like sometimes I have some videos of an exit procedure, so what I discovered, I took videos of an exit procedure previously, it's three or four minutes long showing the procedure, so when we went to the next one I was able to show this video on my phone and say, "This is what's going to happen. This is what the obstetrician will do, this is what the anesthetist will do, this is what the ENT surgeon will do, this is what we're going to do." And so to me, communication briefing and debriefing are really core components of supporting a team, especially new people.” | Interview 10 |
| *Physical Environment (the effect of the physical environment on team coordination)* | “I don't think there's a wrong place to resuscitate, it's just a matter of getting the equipment there. And so, because we've designated the RST room as the resuscitation room and all the equipment is there, then it becomes a problem if you're not going to resuscitate there. Yeah. It is about space.” | Interview 6 |
|  | “I think that the most challenging resus I've ever done was one that was done in the OR. The challenge with that is that you have a very limited space and also a very present audience in terms of like the mom, the obstetrician, the anesthetist, like, and at one point the anesthetist was like over my shoulder. And again, like that can be really challenging cause there are a lot more distractions I feel as far as the environment goes. And then even as far as bringing things in like the crash cart into the OR, like, that's not an easy thing to do. And so you can limit yourself as far as your supplies and your capacity, that way goes. And so as far as teamwork goes, you need more bodies and you need to be more clear about what it is that you need cause to bring things in can be more challenging. Those are all things that like definitely impact how the team functions.” | Interview#8 |
|  | “I think like our best case scenarios are to resuscitate in our RST spaces because we have everything at our disposal. People are familiar with where the equipment is and the everything else, medications, et cetera. So, yeah, just access to equipment, medications, because our crash carts that are in outlying areas aren't as familiar to us. So I think that can be tricky. So to get it done in a timely manner could be like extensive resuscitation could be a bit delayed.” | Interview 9 |
